# Emerging cellular and molecular mechanisms underlying anticancer indications of chrysin

**DOI:** 10.1186/s12935-021-01906-y

**Published:** 2021-04-15

**Authors:** Marjan Talebi, Mohsen Talebi, Tahereh Farkhondeh, Jesus Simal-Gandara, Dalia M. Kopustinskiene, Jurga Bernatoniene, Saeed Samarghandian

**Affiliations:** 1 Department of Pharmacognosy, School of Pharmacy, Shahid Beheshti University of Medical Sciences, 1991953381 Tehran, Iran; 2 Department of Chemistry and Biochemistry, University of Texas at Arlington, Arlington, TX 76019 USA; 3 Food Safety Net Services (FSNS), San Antonio, TX 78216 USA; 4 Cardiovscular Diseases Research Center, Birjand University of Medical Sciences, Birjand, Iran; 5 Faculty of Pharmacy, Birjand University of Medical Sciences, Birjand, Iran; 6 Nutrition and Bromatology Group, Department of Analytical and Food Chemistry, Faculty of Science, University of Vigo, Ourense Campus, 32004 Ourense, Spain; 7 Institute of Pharmaceutical Technologies, Faculty of Pharmacy, Medical Academy, Lithuanian University of Health Sciences, Sukileliu pr. 13, 50161 Kaunas, Lithuania; 8 Noncommunicable Diseases Research Center, Neyshabur University of Medical Sciences, Neyshabur, Iran

**Keywords:** Chrysin, Flavonoids, Oxidative stress, Inflammation, Apoptosis, Metastasis, Proliferation, Transcription factors, Cancer

## Abstract

Chrysin has been shown to exert several beneficial pharmacological activities. Chrysin has anti-cancer, anti-viral, anti-diabetic, neuroprotective, cardioprotective, hepatoprotective, and renoprotective as well as gastrointestinal, respiratory, reproductive, ocular, and skin protective effects through modulating signaling pathway involved in apoptosis, oxidative stress, and inflammation. In the current review, we discussed the emerging cellular and molecular mechanisms underlying therapeutic indications of chrysin in various cancers. Online databases comprising Scopus, PubMed, Embase, ProQuest, Science Direct, Web of Science, and the search engine Google Scholar were searched for available and eligible research articles. The search was conducted by using MeSH terms and keywords in title, abstract, and keywords. In conclusion, experimental studies indicated that chrysin could ameliorate cancers of the breast, gastrointestinal tract, liver and hepatocytes, bladder, male and female reproductive systems, choroid, respiratory tract, thyroid, skin, eye, brain, blood cells, leukemia, osteoblast, and lymph. However, more studies are needed to enhance the bioavailability of chrysin and evaluate this agent in clinical trial studies.

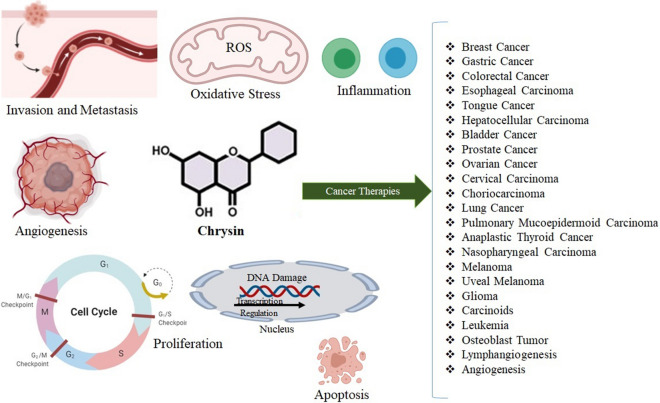

## Introduction

Cancer is the second leading reason for death universally with an appraised of 606,880 deaths in the United States annually. The American Cancer Society (ACS) estimates that 1,762,450 new cases of cancer will be detected in the USA just in 2019 [1]. Hence, discover an approach to treat cancer at numerous stages can assist in rescue people’s lives. Notably, cancers could be treated by combinations of surgical procedures, radiation therapy, chemotherapy, immunotherapy, and hormone therapy [2]. Meanwhile, chemotherapy is one of the most reasonable cures for early- and late-stages. Nevertheless, alopecia, neuropathy, neutropenia, myalgia, nausea, vomiting, diarrhea, fatigue, etc. are the side effects of chemotherapy that lead to less compliance of patients [3]. Flavonoids are considered a versatile source for discovery and development of anticancer agents [4–6]. Chrysin (5,7-dihydroxy-2-phenyl-4*H*-chromene-4-one or 5,7-dihydroxyflavone) is a naturally occurring 15-carbon backbone-based flavonoid [7]. The most reliable pharmacological properties of chrysin are anticancer, neuroprotective, antiviral, antibacterial, antiasthmatic, anti-inflammatory, hepatoprotective, nephroprotective, cardioprotective, anti-diabetic, antidepressant, anxiolytic, and antiarthritic activities [8]. The natural sources of chrysin are honey, where the content of chrysin ranges from 0.10 mg/kg in honeydew honey to 5.3 mg/kg in forest honey [9, 10], propolis (chrysin content 28 g/L and many plants species e.g. *Pelargonium crispum*, *Passiflora incarnate*, *Oroxylum indicum*, *Scutellaria immaculata*, *Scutellaria baicalensis*, *Scutellaria ramosissima*, *Scutellaria discolor, Morinda citrifolia*, *Docynia delavayi*, *Dysphania graveolens*, *Alpiniae oxyphyllae*, *Desmos cochinchinensis*, *Cytisus multiflorus*, *Centaurea omphalotricha*, *Pleurotus ostreatus*, *Indigofera tinctoria*, *Hedyotis diffusa*, *Achyranthes aspera*, *Xylopia pierrei*, and mushrooms *Lactarius deliciosus* (chrysin content ~ 0.17 mg/kg), *Suillus bellinii* (chrysin content 0.34 mg/kg), and a marine endophytic strain called *Chaetomium globosum* [11–26]. In many studies chrysin has been shown to exert beneficial pharmacological activities: it suppressed pro-inflammatory cytokine expression and histamine release, downregulated nuclear factor kappa B (NF-kB), cyclooxygenase 2 (COX-2), and inducible nitric oxide synthase (iNOS) [27], upregulated apoptotic pathways [28], inhibited angiogenesis [29] and metastasis formation [30] protecting from cancer, suppressed DNA topoisomerases [31] and histone deacetylase [32], downregulated tumor necrosis factor α (TNF-α) and interleukin 1β (IL-1β) [33], promoted protective signaling pathways in the heart [34], kidney [35] and brain [8], decreased cholesterol level [36], and demonstrated a potent anti-glycemic activity [37]. Regarding these vulnerable potentials of chrysin in the prevention and treatment of physiological disturbances, we reviewed feasible cellular and molecular mechanisms involved in the anticancer impacts of chrysin.

### Chemical Properties of chrysin and its derivatives

**Fig. 1:**
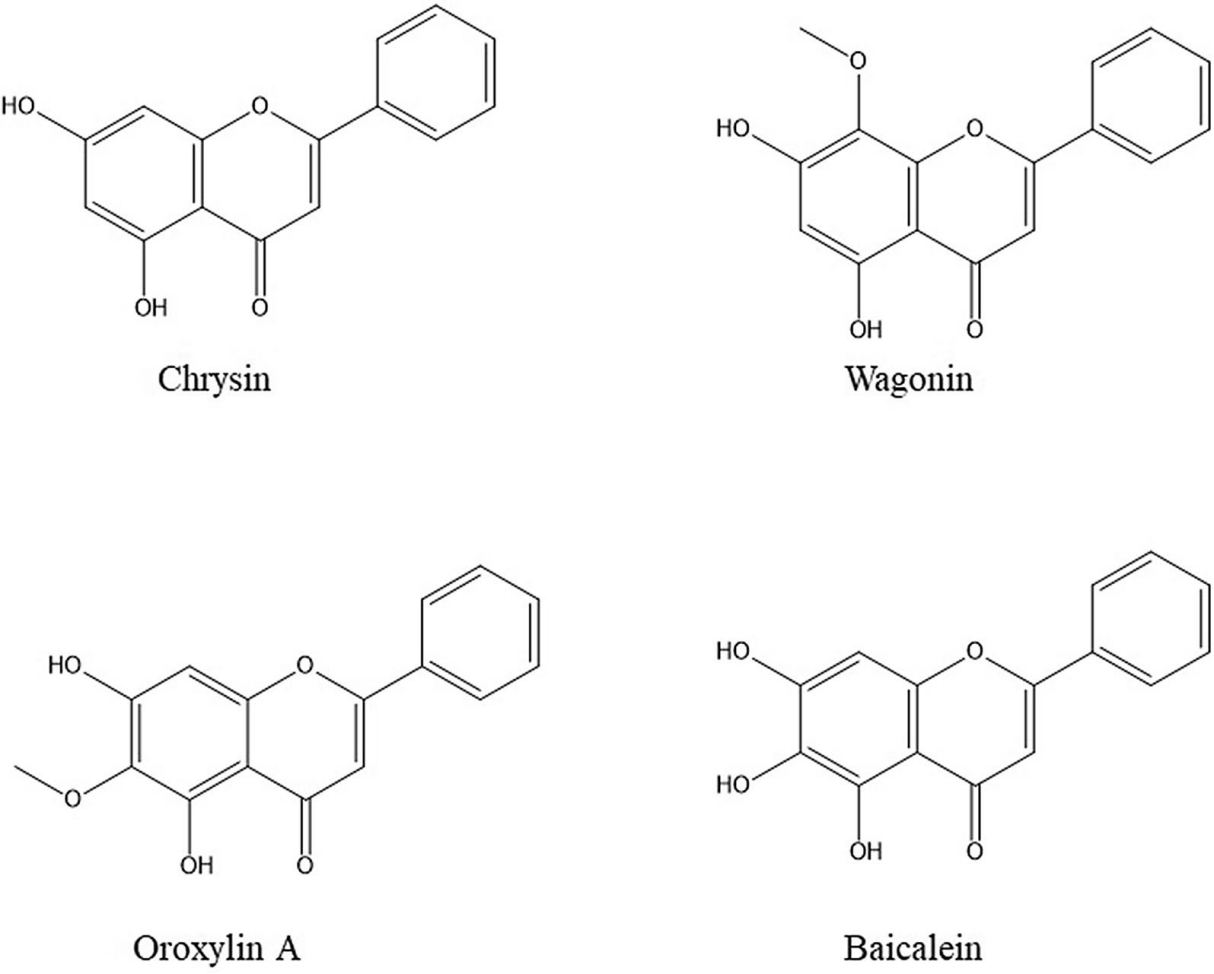
Chrysin chemical structure

Chrysin has two benzene rings (A and B) and an oxygen-containing heterocyclic ring (C) in its structure [38]. The antioxidant activity of chrysin is related to the presence of the double bond between C2–C3 and the carbonyl group on the C4 atom [39–41]. Differently, from many flavonoids with –OH groups on C3 and C4 atoms in ring B, chrysin lacks oxygenation in B and C rings, and this structural property is linked to the main biological activities of chrysin, ranging from the anti-inflammatory to antitoxic effects [39, 40]. There are –OH groups at C5 and C7 atoms in chrysin structure, related to the free oxygen radical scavenging activities [39, 40]. The diversity in the ring-A oxygenation is the principal reason for the formation of numerous natural derivatives of chrysin like baicalein, Oroxylin A, and wogonin [8] (Fig. 1). To enhance the biological activity of chrysin, its various derivatives were synthesized, introducing different substituents in its molecule [42]. The introduction of hydrophobic chains at C5 and C7 positions improved chrysin’s anti-inflammatory activity [43]. The C30, C40-dichloro substituent in the chrysin molecule was responsible for the suppression of prostaglandin (PG) production [44]. Using chrysin backbone following attachment of nitric oxide donor pro-drugs promoted vasculoprotective activity [45] and angiogenesis [46]. Hexadecyl 2-(5-hydroxy-4-oxo-2-phenyl-4*H*-chromene-7-yloxy) acetate and *N*-hexadecyl 2-(5-hydroxy-4-oxo-2-phenyl-4*H*-chromen-7-yloxy) acetamide were exerted antiproliferative effect [47]. Antitumor activity by 5,7-diacetyl chrysin has been reported in H22 cells in vitro [48]. Diethyl chrysin-7-yl phosphate and tetraethyl bis-phosphoric ester of chrysin were obtained by phosphorylation of –OH groups at C7 or both C5 and C7 atoms resulting in the enhancement of chrysin antiproliferative properties [49]. Methylation of both C5 and C7 resulted in higher effectiveness of this chrysin analog as a feasible chemotherapeutic agent for acute lymphoblastic leukemia [50]. Butyl, octyl, propyl, and tolyl derivatives of the C5- and C7-hydroxyl groups were linked to the anti-glycemic effect deprived of side effects up to 500 mg/kg [51]. By in silico screening, a series of C7-hydroxyproton substituted chrysin derivatives exhibited EGFR inhibiting possessions against breast cancer [52]. Fluorine-containing chrysin derivatives showed greater antimicrobial and anticancer effects [53].

**Fig.2:**
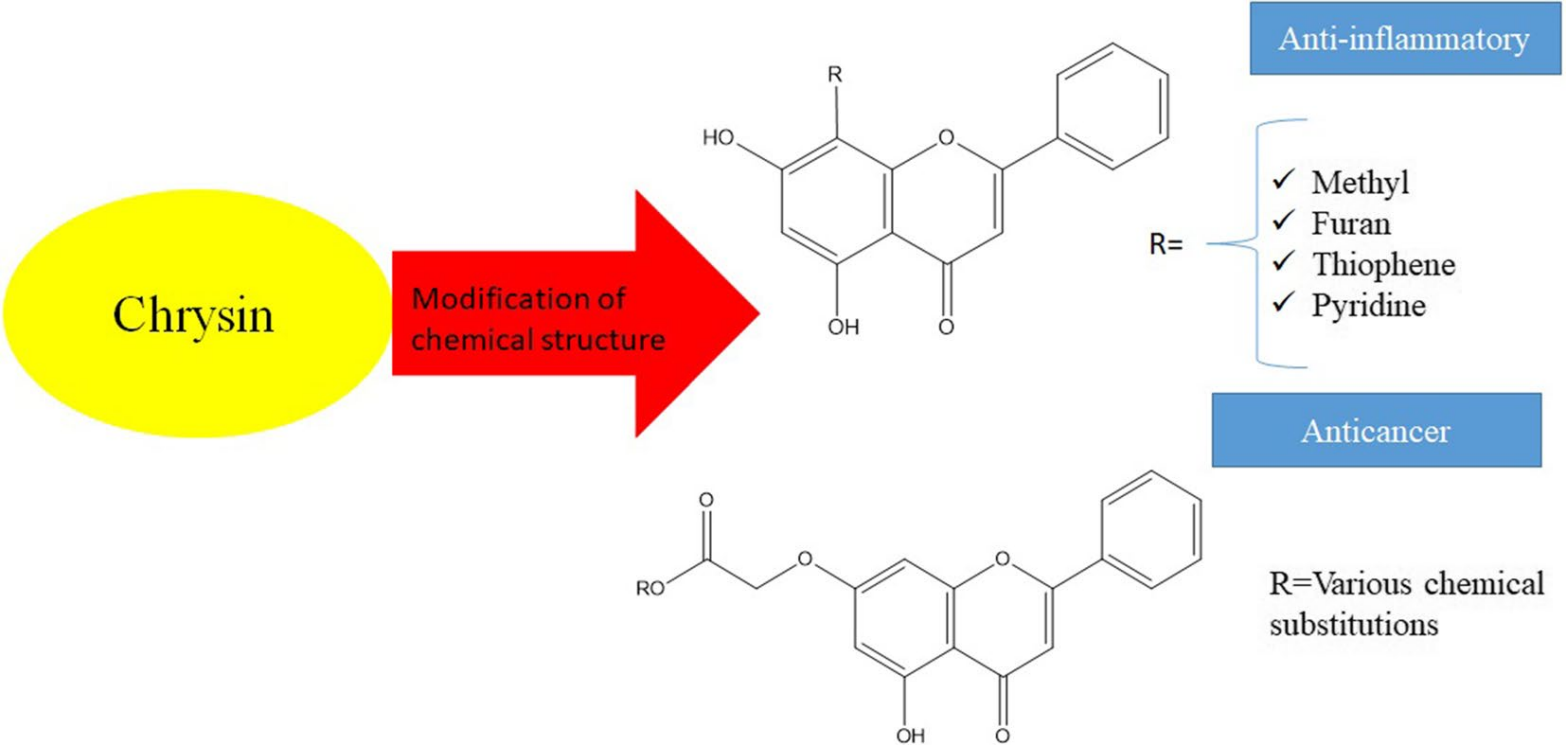
Association of structural modification of chrysin and its substituent pharmacological effects

In a series of chrysin-benzothiazole conjugates using 7-(4-bromobutoxy)-5-hydroxy-2-phenyl-4*H*-chromene-4-one, the presence of halogens, the length of the aliphatic side sequence linking two different pharmacophores as chrysin and benzothiazole, the characteristics and site of the electron-withdrawing and electron-donating functional groups on the benzothiazole backbone were related to the increase in the anticancer activity of chrysin [54] (Fig. 2).

### Bioavailability, drug delivery, and toxicity of chrysin

In human organism chrysin is poorly absorbed, rapidly metabolized, and eliminated, therefore its bioavailability is very low [55]. Chrysin is metabolized via conjugation reactions, mainly sulfation and glucuronidation, and less via oxidation in intestinal and hepatic cells [56]. Chrysin sulfonate and glucuronide were existing in the urine and plasma at low concentrations [56], the highest concentrations of chrysin sulfate and glucuronide were observed in the bile in the studies of chrysin metabolism in mice [57], thus the excretion through feces is the main suggested way for the elimination of chrysin and its metabolites [8, 56, 57]. There was reported very low concentrations of this flavonoid in plasma afterward a single 400 mg oral dose of chrysin in humans, the plasma binding was > 99% [55]. The bioavailability of chrysin in the oral route of administration was appraised to be 0.003–0.02% [55], the maximum plasma concentration—12–64 nM [58]. The predicted maximum serum concentration for flavonoid aglycones, generally, is 1 mmol/L [55], thus the chrysin should be administered to reach the serum concentration in the micromolar range [42]. Drug delivery systems for chrysin using nanoparticles, liposomes, and micelles as carriers have been reported [59–62]. The best approach to overcome the poor bioavailability of chrysin was its encapsulation in nanoparticles [63].

Entrapping chrysin in niosomal oromuco-adhesive films is one of the great strategies to gain a therapeutic drug delivery platform in order to combat oral recurrent aphthous ulcers and enhance the bioavailability of chrysin [64].

Encapsulation of chrysin in to poly (d, l-lactic-*co*-glycolic acid) poly (ethylene glycol) (PLGA-PEG) nanoparticles controlled the release kinetic of chryisn and its cytotoxicity [65]. It has been found that chrysin/curcumin loaded in PLGA-PEG reduced the expression of cyclin D1 and the proliferation of breast cancer cells [59]. Moreover, PLGA-PEG nanoparticles endorsed anticancer effects of curcumin/chrysin by attenuation of hTERT gene expression in colorectal cancer SW480 cells and resulted in elevation of bioavailability and the solubility of these naturally-based medications [66]. In addition, chrysin-encapsulated to nanopolymer enhanced the efficacy of chemotherapeutic agents. In this context, previous study indicated that doxorubicin was loaded to PEG-chrysin conjugate nonparticle has more efficacy and safety [67]. It was also found that chrysin delivery with nanostructured lipid carriers (NLCs) increased the efficacy of doxorubicin via decreasing the activity of drug efflux pumps and detoxification enzymes [68]. Doxorubicin loaded mPEG–PCL–chrysin micelles could significantly show potent anticancer activities in vitro, regarding the π–π stacking interactions among the mentioned micelles and doxorubicin [69]. P(HEMA-LA-MADQUAT) micelles could assist in co-delivery of methotrexate and chrysin in co-treatment approach in MCF-7 cells [70].

Chrysin has been shown to boost testosterone in humans [71]. The adverse effects have not been reported at the daily dosage of 400–500 mg of chrysin [72]. The suggested amount of chrysin to be consumed daily is considered to be 0.5–3 g [8, 55, 73]. While low doses of flavonoids are available in the regular dietary intake of individuals, consumption of higher doses may initiate toxicity [8, 73]. Chrysin has been reported to induce toxicity in trout liver cells and suppression of de novo DNA formation, causing abridged cell numbers [40]. The cytotoxicity due to chrysin has been credited to the possession of peroxidase-like activity in hepatocytes, inducing chrysin’s oxidation, consequently making toxic agents. Myeloperoxidase and topoisomerase II also were linked to the toxic effects of chrysin [40], 74.

## Literature search methodology

Online databases comprising Scopus, PubMed, Embase, ProQuest, Science Direct, Web of Science, and the search engine Google Scholar were searched for available and eligible research articles. The search was conducted by using MeSH terms in title, abstract, and keywords. Obtained results were screened by two independent authors. Studies in English (abstract/full text), original research, with full text available, and showing preventive or therapeutic aspects of chrysin by focusing on involved cellular and molecular mechanisms were included. Data of the literature search was extracted and reported in the current review article (Table 1).

**Table 1:** Some of the molecular and cellular mechanisms involved in preventive and therapeutic indications of chrysin

Health effect	Mechanisms	Refs.
Breast cancer	Antiproliferative effect	[79]
Breast cancer	Downregulated cyclin D1 and hTERT	[78]
Breast cancer Stem cell	Inhibited EGFR	[84]
Breast cancer	Apoptosis	[85]
Breast cancer	Apoptosis	[80]
Breast cancer	Inhibited angiogenesis, alleviated VEGF expression, Suppressed metastatic growth due to alleviation of activation of STAT3 and hypoxic survival	[30]
Breast cancer	Inhibited of HDAC8 enzymatic activity	[213]
Breast cancer	modulated phase I and phase II enzymes	[214]
Gastric cancer	Altered microRNAs expression	[94]
Gastric cancer	Blocked AP-1 and suppressed early growth response-1	[98, 99]
Buccal pouch carcinoma	mitigated phase-I enzymes (Cyt b5 and Cyt p 450) and increased phase-II enzymes (GSH, GST, GR, and DTD)	[215]
Colon cancer	Arrested G2/M phase of cell cycle	[112]
Colorectal cancer	Inhibited cell proliferation, improved antioxidant mineral levels, reduced nitrosative stress	[216]
Colon cancer	Modulated cryptal cell proliferation activity inhibited apoptosis	[217]
Hepatocellular carcinoma	Overexpressed hexokinase-2	[129]
Hepatocellular carcinoma	attenuated NF-kB p65 levels and COX-2 expression, reduced Bax, Bcl-xL, β-arrestin-2, caspase-3, and p53 regarding apoptosis	[136]
Hepatocellular carcinoma	Attenuated the canonical Wnt and NF-kB, induced apoptosis	[140]
Liver cancer	Downregulated the β-catenin expression	[218]
Renal carcinoma	Ameliorated oxidative stress, hyperproliferation, and inflammation through NF-kB pathway	[143]
Skin cancer	Attenuated the MSK1/histone H3 signaling	[219]
Skin cancer	Inhibited tumor growth and neoplastic transformation by targeting CDK2 and CDK4	[220]
Melanoma	mitigated the TERT, MMP-2, and MMP-9 genes levels, ameliorated genes expressions of TIMP-1 and TIMP-2	[192]
Anaplastic thyroid carcinoma	Induced apoptosis by activating Notch1 signaling related to PARP cleavage	[180]
Prostate cancer	Inhibited expression of HIF-1α via Akt signaling pathway and abrogated VEGF expression	[153]
Prostate cancer	Inhibited DNA methyltransferases	[221]
Leukemia	Enhanced populations of T-and B cells (CD-3, CD-19, and Mac-3), Promoted macrophage phagocytosis and NK cell cytotoxicity	[202]
Leukemia	reduced cell viability and induced DNA fragmentation regarding apoptotic cell death	[222]
Leukemia	Induced apoptosis in Bcl-2 overexpressing associated with PLC-ϒ1 degradation, caspase-3 activation, XIAP downregulation, and the Akt inactivation	[205, 223]
Leukemia	attenuated SCF/c-Kit signaling by abrogation of PI3K pathway	[204]
Cervical cancer	inhibited proliferation and induced apoptosis	[49]
Cervical cancer	induced p38 and NF-kB/p65 activation	[224]
Cervical cancer	Increased caspases-3 and -9, Bax, and cleaved-PARP expression, caused arrest in G2 phase of cell cycle	[22]
Cervical and ovarian cancer	Antioxidant and anticancer	[54]
Ovarian cancer	Antioxidant and anticancer	[225]
NSCLC	Inhibited IL-6-induced AKR1C1/1C2 overexpression	[176]
Glioma	Antiproliferative and apoptotic activity	[194]
Glioma	Increased accumulation of arsenic	[226]
Ehrlich ascites	Enhanced functional activity of macrophages	[212]

## Cellular and molecular mechanisms involved in the therapeutic effects of chrysin

### Anticancer effects of chrysin

#### Breast cancer

Breast cancer is an important reason of deaths in women globally [75]. Chrysin pretreatment increased p53 protein expression and diminished viability of MCF7 cells. Pretreatment with chrysin also activated the ATM-Chk2 pathway without DNA damage [76]. In a previous study coadministration of chrysin and metformin, against T47D breast cancer cells were examined. T47D cells were treated with Metformin, Chrysin, and their combination. Chrysin alone and potentially in combination with metformin decreased cyclin D1 and hTERT gene expression in the T47D breast cancer cell line [77, 78]. In a study of the MCF-7 cell line, induction of cell apoptosis was obtained by chrysin pretreatment [79]. Yang et al. utilized metastatic triple-negative breast cancer (TNBC) cell lines to investigate the antimetastatic effect of chrysin. Chrysin pretreatment inhibited MMP-10 and Akt signaling pathways [80]. Lirdprapamongkol and coworkers studied on 4T1 murine mammary carcinoma cell line in hypoxic conditions and xenografts. Chrysin declined hypoxic survival, inhibited activation of STAT3, and reduced VEGF expression in hypoxic cancer cells, causing annulment of hypoxia-induced angiogenesis and occasioning suppression of metastatic growth [30]. Chrysin ameliorated TRAIL-mediated apoptosis in MDA-MB-231 [81]. Hepatic microsomes from Nile tilapia (*Oreochromis niloticus*) were investigated. Chrysin inhibited the MCF-7 cancer cells proliferation and also it had powerful anti-aromatase activity [82]. MDA-MB-231 cells were utilized for the evaluation of chrysin effects. Chrysin induced apoptosis via inducing Skp2 and LRP6 expressions. Chrysin pretreatment also downregulated MMP2, MMP9, fibronectin, and snail expression. The mRNA expression of PPARα noticeably increased in chrysin-treated MDA-MB-231 cells, which was feasibly related to the anti-proliferative effects of chrysin [17, 83]. The capability of an analog of chrysin to inhibit EGFR was reported in a breast cancer stem cell model [84]. A novel chrysin-organotin (Chrysin-Sn) compound considerably elevated ROS levels in MCF-7 cells and regarding apoptosis activated caspase-3 and prompted autophagy through augmentation of LC3-II level [85]. Chrysin-ruthenium complex modulated mTOR, VEGF, and p53 signaling pathways in the MCF-7 cells [86]. Pretreatment with chrysin remarkably suppressed TNBC cell migration and invasion. chrysin downregulated MMP-10, reduced snail, slug, and vimentin expressions increased E-cadherin expression, and inhibited Akt signaling pathway in TNBC cells, proposing that chrysin possessed a reversal activity on EMT [80]. Zhang and coworkers found that pretreating MCF-7 cells with chrysin caused BCRP inhibition, which was resulted in significant increases in mitoxantrone accumulation [87]. 8-bromo-7-methoxychrysin, a synthetic analog of chrysin exhibited anticancer properties via downregulation of CDK4, cyclin D1, and cyclin E, inactivation of Akt, GSK-3β and β-catenin in HER-2/neu-overexpressing MDA-MB-453 and BT-474 cells [88]. Zhao and coworkers found that 5,7-dihydroxy-8-nitrochrysin could stimulate cell fate in MDA-MB-453 cells via activation of caspase and modulation of the Akt/FOXO3a pathway [89]. By substitution of benzyloxy, dimethylamino, nitro, and fluoro on chrysin structure, potent cytotoxic agents were synthetized that displayed considerable cytotoxicity against MDA-MB-231 and MCF-7 [90].

Fabrication of chrysin-attached to silver and gold nanoparticles crossbred reduced graphene oxide nanocomposites led to augmentation of the generation of ROS-induced apoptosis in breast cancer [91]. Chrysin loaded PCL-PEG-PCL had greater antitumor impact on gene expression of BRCA1, FTO, and hTERT in comparison to free chrysin [92].

#### Gastric cancer

Gastric cancer is considered as the third important reason of cancer-related deaths globally. Various etiologies have participated in the initializations and progressions of gastric cancer including gene-environment dealings with *Helicobacter pylori* as the most prevailing reasons for the pathogenesis of gastric cancer, numerous genetic and epigenetic changes have been connected with its carcinogenesis moreover. The human gastric epithelial cell line (GES-1) and human gastric cancer cell lines (MKN-45) and Nude Mice Xenograft Model were studied. Evaluation of Ten-eleven translocation (TET) 1 expression via qRT-PCR following chrysin consumption was assessed. Chrysin induced augmentation in TET1 (responsible for cell apoptosis, migration, and invasion) expression via enhancement of 5hmC levels and exerted cytotoxic effects in MKN-45 cells. Chrysin-treatment caused inhibition of cell migration and attenuation of invasion in MKN-45 cells. Cell apoptosis (Bax and Bcl2) and cell cycle altered by G0/G1 arrest and decline in the number of cells in the S phase. From in vivo findings, it was concluded that chrysin reduced tumor growth and promoted TET1 expression. CRISPR/Cas9 system was used to generate the TET1 gene knocked out. Collectively, the study directed that chrysin displayed anti-tumor properties through regulating TET1 expression [93]. In another study, it has been observed that chrysin downregulated miR-18, miR-21, and miR-221 expression however upregulated let-7a, miR-9, miR-22, miR-34a, and miR-126 expression in the gastric carcinoma cell line [94]. Higher elevation of miR-22, miR-34a, miR-126, miR-9 and Let-7a gene expression was observed in the case of utilization of chrysin-PLGA-PEG nanoparticles, in comparison with free chrysin [95, 96]. Higher downregulation of miR-18a, miR-21 and miR-221 genes was obtained by chrysin-loaded PLGA-PEG nanoparticles [97]. The AGS human gastric cancer cell line was utilized in a study. Chrysin controlled MMP-9 expression via suppression of AP-1 activity which blocked ERK1/2 and JNK1/2 signaling pathways in gastric cancer AGS cells [98]. Chrysin significantly inhibited endogenous and inducible Recepteur d’origine Nantais (RON) expression. Chrysin inhibited Egr-1 and NF-κB transcription factor activities in AGS [99]. Bakhsheshian and coworkers demonstrated that chrysin inhibited Pp-18 efflux in both human and mouse ABCG2 [100]. A chrysin benzimidazole derivative could arrest the G0/G1 phase of the cell cycle in MFC cells [101]. Ai and coworkers used SGC-7901 cells to evaluate the efficacy of 5, 7-dihydroxy-8-nitrochrysin (NOChR). NOChR induced apoptosis of SGC-7901 cell lines by activation of PPARγ and reduction of the Bcl-2/Bax ratio [102]. Chrysin overcomed the 5-FU-resistance in gastric cancer AGS and AGS/FR cells via S phase arrest [103]. 8-Bromo-7-methoxychrysin induced apoptotic cell fate in SGC-7901 cell line partly by motivating PPARγ [104]. 7-*O*-carboxymethyl chrysin exhibited greater apoptotic and anti-proliferative effects on human gastric carcinoma MGC-803 cells [105].

#### Colorectal cancer

Colorectal cancer is one of the most globally common types of cancers with continuously increased incidence each year [106]. Chrysin meaningfully amplified LC3-II levels, an autophagy-associated marker, in colorectal cancer cells. Pretreatment with chrysin induced ROS formation, and consecutively, inhibited Akt phosphorylation and mTOR. In accumulation, the reported findings suggested that chrysin might be a potential candidate through autophagy which can be replaced 5-FU and oxaliplatin combination combat colorectal tumors for colorectal cancer management in the coming [107]. In earlier research, it has been revealed that AHR was mandatory for the apoptosis inducting following pretreatment with chrysin. The augmentation of TNF-α and TNF-β gene expression in human colorectal cancer cells were found [108]. In vivo transplanted CT26 tumor cells in mice and in vitro CT26 cells were investigated. Chrysin reduced tumor volume via the upregulation of the Bax and downregulation of the sall4 [109]. Chrysin ameliorated TRAIL-mediated apoptosis in HT-29 and HCT-116 cell lines [28, 81]. Human colon carcinoma cells (Caco-2) were utilized by Schumacher and coworkers. Pretreating cells with chrysin inhibited P-gp, MRP-2, and BCRP. Moreover, chrysin augmented ABC-transporters expression in Caco-2 cells [110]. Romier et al. found that chrysin intensely abridged IL-1β-induced IkB-α phosphorylation, diminished IL-8 secretion, and blocked NF-kB activation via the inhibition of IkB-α phosphorylation [111]. The pretreatment of HT-29 with chrysin oxidovanadium (IV) complex evoked cell cycle arrest in the G2/M phase [112]. Effects of chrysin on HCT116 colon adenocarcinoma cell lines were studied. Chrysin inhibited mRNA expression of PPARα, significantly increased cell population of the G0/G1 phase, and declined the proportion in S phase. Hence chrysin regulated the migration activity and the expression of CYP1B1 and CYP2S1 in colorectal cancer cells [113]. Galijatovic et al. found that chrysin pretreatment increased UGT1AI expression in Caco-2 cells [114]. Encountering of HCT-116 cells with chrysin resulted in DNA damage and prompted mitochondrial membrane agitation go along with downregulation of Bcl-2, activation of BID and Bax, cytochrome c release, and caspase-3-mediated apoptosis. Regarding the aforementioned findings, ROS production by chrysin was the critical mediator behind induction of ER stress, leading to JNK phosphorylation, intracellular Ca^2+^ release, and activation of the mitochondrial apoptosis pathway [115].

Salama et al*.* discovered that anticancer activity of chrysin against SW620 cells were connected with reduced protein expression of p-ERK/ERK and p-Akt/Akt [116]. Ren and coworkers found that 7-piperazinethylchrysin alleviated mitochondrial membrane potential of HCT-116 cells and augmented the generation of intracellular ROS. In addition, elevation of Bax and reduction of Bcl-2 at protein expression levels were observed. Activation of p53, caspase-3 and -9, release of cytochrome c, PARP1 cleavage, and downregulation of p-Akt were other alterations following the use of 7-piperazinethylchrysin [117].

#### Esophageal carcinoma

Squamous cell carcinoma and esophageal malignancies are the two main types of esophageal cancer, with distinct etiological and pathological features. Two reports indicated that chrysin exhibited cytotoxicity in human esophageal squamous cell carcinoma cell lines (KYSE-510) and (OE33). It was observed that the treatment of KYSE-510 and OE33 cells with chrysin inhibited the G2/M cycle via the up-regulation of P21 and GADD45-beta and down-regulation of cyclin B1 at the mRNA and protein levels. Besides, chrysin induced p53-independent mitochondrial-mediated apoptosis via up-regulation of PIG3 and cleavage of caspase-9 and -3 [118–120]. Downregulation of cyclin B1 and cyclin D1 and upregulation of 14-3-3σ at the mRNA and protein levels which were related to the proliferation and differentiation of cells were witnessed afterward the treatment of OE33 cells with chrysin [121, 122].

#### Tongue cancer

The squamous cell carcinoma (SCC) is frequently observed in the oral cavity that is highly invasive with high lymph nodes metastases. It was found that proline metabolism and proline dehydrogenase/proline oxidase (PRODH/POX) has the main role in the modulation of cancer cell survival/apoptosis. To assess the effect of chrysin on the cytotoxicity, proliferation, expression of apoptotic protein and proline metabolism and concentration in SCC, the MTT, proliferation and western blot assays and also HPLC were used, respectively. Chrysin could stimulate anti-proliferative activity, as well as the expression of PRODH/POX, P53, caspases-3 and -9 and reduced collagen biosynthesis, prolidase activity, and proline concentration in human tongue squamous cell carcinoma (CAL-27) cells. Indeed, chrysin induced PRODH/POX-dependent apoptosis via an increase in the degradation of mitochondrial proline and a decrease in proline content for collagen biosynthesis [123]. Xie and colleagues demonstrated that chrysin had apoptotic effects on KB cells which might be associated to mitochondrial dysfunction and hindering of PI3K/Akt cascade [124].

#### Hepatocellular carcinoma

Hepatocellular carcinoma is one of the most communal gastrointestinal system malignancies, place as the fifth fatal cause of cancers universal [125]. SMMC-7721 and MHCC97H cells were evaluated for the determination of chrysin potential to combat hepatocellular carcinoma. Chrysin meaningfully inhibited sphere formation and upregulated SHP-1 protein expression in SMMC-7721 and MHCC97H cells, besides abridged p-STAT3 and Twist1 expressions in SMMC-7721 cells. collectively, it has appeared that chrysin acted as a nominee against HCC via regulating the SHP-1/STAT3 signaling pathway [126]. Sorafenib is a multikinase inhibitor as a proven treatment for progressive HCC. Conversely, its therapeutic efficacy is not as worthy as was expected. Therefore, improvement sensitivity of HCC to sorafenib would be effective. A previous study verified that coadministration of chrysin improved sorafenib sensitivity through inhibition of ATP-binding cassette superfamily G member 2 (ABCG2). Hep3B and HepG2 HCC cells were assessed. Chrysin prompted sustained ERK1/2 phosphorylation and promoted overexpression of mitogen-activated protein kinase 1 (MEK1). These findings displayed the ERK1/2 phosphorylation mechanism contributing a chrysin-mediated synergistic effect on sorafenib sensitivity in HCC cells [127]. In another study, 2-acetylaminofluorene (2-AAF) and diethylnitrosamine (DEN) were used for the induction of HCC in rats. Chrysin pretreatment led to an increase in mitochondrial ROS creation, swelling in isolated mitochondria from hepatocytes, collapse in MMP, and release cytochrome *c*. Furthermore, Chrysin could elevate caspase-3 activity in the HCC rats group. From these findings, chrysin could be considered as a talented complementary therapeutic candidate combat HCC, but further preclinical and clinical trials are needed [128]. In a previous study of HCC cells and xenograft models, chrysin declined HK-2 combined with VDAC-1 on mitochondria, resulted in the transformation of Bax to mitochondria and induced cell apoptosis [129]. The effects of chrysin in human HCC, QGY7701, and HepG2 cells were evaluated in a recent study. Chrysin encountering improved proapoptotic protein expression, containing Bax, Bad, Bak, and p53 whereas it reduced Bcl-2. It was revealed that chrysin motivated programmed cell death in the HCC cells by modulating the p53/Bcl-2/caspase-9 signaling pathway [130, 131]. Chrysin arrested the SubG0 phase of the cell cycle in HepG2 cells [132]. Chrysin ameliorated TRAIL-mediated apoptosis in HepG2 [81]. Gao et al. elucidated that a higher level of Nrf2 expressed in BEL-7402/ADM cells associated with doxorubicin resistance, and chrysin inhibited the Nrf2 expression and its downstream genes comprising AKR1B10, HO-1, and MRP5 by quenching ERK and PI3K-Akt pathway and ultimately resulted in a reversal of drug-resistant phenotype [133, 134]. H22 ascitic hepatoma cells and xenograft mice were exposed to chrysin. Chrysin activated caspase-3 regarding apoptosis, but also inhibited the generation of VEGF and suppressed angiogenesis [11]. Hepatic microsomes from Nile tilapia (*Oreochromis niloticus*) were investigated. Chrysin inhibited the proliferation of HepG2 cancer cells and also it had powerful anti-aromatase activity [82]. Huang et al*.* demonstrated that, chrysin induced apoptosis via inducing Skp2 and LRP6 expressions. Chrysin pretreatment also downregulated MMP2, MMP9, fibronectin, and snail expression [17]. Sun et al. demonstrated that chrysin induced GRP78 overexpression, spliced XBP-1, and eIF2-α phosphorylation. Besides, Chrysin persuaded caspase-7 cleavage and PARP cleavage [135]. Khan and coworkers revealed the effectiveness of chrysin in DEN-induced early hepatocarcinogenesis in rats. Chrysin administration significantly reduced AST, ALT, ALP, LDH and γGT serum activities. Moreover, chrysin attenuated COX-2 and NFkB p65 expression, and Bcl-xL and β-arrestin levels, whereas that of p53, Bax and caspase 3 increased at the mRNA and protein levels [136]. Walle et al*.* revealed that chrysin induced UDP-glucuronosyltransferase UGT1A1 in HepG2 cells [137]. Sherif et al*.* discovered that chrysin could combat hepatocellular carinoma through the inhibition of the GPC3/SULF2 axis accompanied by the downregulation of lncRNA-AF085935 expression [138]. Wang and collegues found that chrysin nanosuspension had higher anti-tumor effects against human HepG2 cells [139]. Administration of methylated chrysin in the early hepatocarcinogenesis rat model resulted in attenuation of Wnt and NF-kB pathways [140]. Yang et al*.* observed that 8-bromo-7-methoxychrysin could induce apoptosis of HepG2, Bel-7402 and L-02 cells by generation of ROS and sustained activation of JNK [141].

#### Renal cell carcinoma

Renal cell carcinoma (RCC) is one of the most common malignancies in adults’ kidneys. RCC is regularly resistant to conventional chemotherapeutic regimens. A rat model of renal cancer was initiated by DEN and promoted by ferric nitrilotriacetate (Fe-NTA). Administration of chrysin alleviated LPO and promoted CAT, GSH, GR, and GPx activities. Moreover, chrysin diminished BUN and creatinine. Reduction in IL-6 and TNF-α and augmentation in caspases-9 and 3 were observed due to chrysin supplementation. Chrysin induced entire suppression NF-kB, COX-2, PG-E2, iNOS as well. Downregulation of PCNA, ODC and Bcl-2 vice versa upregulation of Bax proteins have resulted in chrysin supplementation [142, 143].

#### Bladder cancer

Bladder cancer is the second most communal type of ‘tract cancer’ in developed countries.

One study investigated the effect of chrysin on apoptosis, ROS production and DNA fragmentation by using western blot and flow cytometer techniques. Chrysin provoked apoptosis due to activation of caspases- 3 and 9, reduced Bcl‑2, Mcl‑1, Bcl‑xl expression, and promoted Bax protein expression. Chrysin also persuaded ER stress via activation of the unfolded protein response of PRKR‑like ERK, eIF2α, and activating transcription factor 4 in bladder cancer cells, and inhibited the signal transducer and activator of the transcription 3 pathway. Furthermore, the alleviation of ROS generation was detected following treatment with chrysin [144]. The results suggested that chrysin was effective against bladder cancer through increasing apoptosis and ROS production. One of the main markers indicated the poor prognosis in patients with urinary bladder tumor that is mutation in tumor protein p53 (TP53) gene. It was found that the progression of bladder tumor cell was inhibited by chrysin at doses 10–100 µM in mutated and wild type TP53 in grade 1–3. Cell proliferation inhibition by chrsin was confirmed by elevation in reactive oxygen species (ROS) production and decrease in DNA damage. Chrysin could affect cell cycle at G2 and M phases and cell morphology following decrease in the expression of PLK1, HOXB3 and SRC genes in mutated TP53 cells,. Chrysin also stimulated in DNA hypermethylation grade 2 cells, and decreased the expression of c-MYC, FGFR3 and mTOR gene in grade 3 cells. The authors suggested that the anti-proliferative effect of chrysin was not dependent to TP53 status in bladder tumor cells; but, the involved mechanisms are associated with TP53 status [145, 146]. Szliszka and coworkers observed that combination-therapy of bladder cancer cells with TRAIL and chrysin led to higher sensitization of bladder cancer cells to TRAIL prompted cytotoxicity [147, 148].

#### Prostate cancer

Prostate cancer is a frequently diagnosed cancer in men worldwide [149]. Prostate cancer (DU145 and PC-3) cell lines were used to assess the role of chrysin in prostate cancer. Chrysin induced apoptosis of cells by causing DNA fragmentation and increasing the proportions of DU145 and PC-3 cells in the sub-G1 phase of the cell cycle. Additionally, chrysin abridged the expression of proliferating cell nuclear antigen in the DU145 and PC-3 cell lines. Likewise, chrysin induced loss of mitochondria membrane potential, while augmented lipid peroxidation and ROS production. Also, it induced ER stress via activation of UPR proteins comprising PERK, eIF2α, and GRP78 in DU145 and PC-3 cells. The chrysin-mediated intracellular signaling pathways suppressed PI3K and the abundance of AKT, S6, P70S6K, and P90RSK proteins, but motivated MAPK and activation of P38 and ERK1/2 proteins in the prostate cancer cells [150–152]. Fu and coworkers utilized DU145 cells and DU145 xenograft-induced angiogenesis in nude mice. Chrysin inhibited insulin-induced expression of (hypoxia-inducible factor-1α) HIF-1α by decreasing its stability. Chrysin increased the ubiquitination and degradation of HIF-1α by increasing its prolyl hydroxylation. Furthermore, chrysin hindered with the interaction between HIF-1α and heat shock protein 90. Chrysin abrogated HIF-1α expression through AKT signaling, which led to the suppression of VEGF expression [153]. Szliszka and coworkers elucidated the higher sensitization of LNCaP cells through upregulation of TRAIL-R2 following the chrysin pretreatment [154].

#### Ovarian cancer

Coadministration of TNF-α and TGF-β with or without the presence of chrysin was studied in OVCAR-3 cells. For this reason, proliferation assays (using a cell proliferation enzyme-linked immunosorbent assay (ELISA) 5-bromo-2′-deoxyuridine (BrdU) kit), immunofluorescence microscopy (the expression of proliferating cell nuclear antigen (PCNA), flow cytometer (ROS production), mitochondrial staining kit (mitochondrial membrane potential) and Western blot analysis (protein expression) were used. Chrysin inhibited a pro-inflammatory cytokine to induce EMT and CSLC features in OVCAR-3 cells, which may be convoluted in hindering the NF-κB/Twist axis [155]. Chrysin’s role in the progression of (ES2 and OV90) ovarian cancer cell lines was studied. Chrysin pretreatment increased ROS formation, cytoplasmic Ca (2+) levels, and diminished mitochondrial membrane potential (MMP). Furthermore, the chrysin activated MAPK and PI3K/AKT pathway [156, 157]. Chrysin inhibited the sphere formation ability of SKOV3-derived ovarian cancer stem-like cells through downregulation of CK2α protein expression [158]. 8‑bromo‑7‑methoxychrysin led to cell fate in cisplatin‑sensitive/resistant A2780 and A2780/DDP cells happened via the regulation of Akt/FOXO3a, resulting in transcription of Bim [159]. Selenium–chrysin polyurea dendrimer nanoformulation increased GSH depletion cystathionine β-synthase inhibition in ES2, OVCAR3, and OVCAR8 cell lines [160]. 5-Allyl-7-Gen-Difluoromethylenechrysin induced apoptosis in CoC1 cells by activation of PPARγ accompanied by alleviation of protein levels of NF-κB and Bcl-2 and augmentation of Bax expression [161].

#### Cervical carcinoma

Cervical carcinoma can be induced following chronic infection with high-risk human papillomavirus (HPV) and is one of the reasons for fatal malignancies [162, 163]. HeLa cell line was studied to investigate the effects of chrysin co-administered by TNF-α and TGF-β. HeLa cells were exposed to TNF-α and TGF-β for one day after TGF-β alone for 6 d with or whit out chrysin at 5.0, 10.0 and 20.0 μM concentrations. The levels of EMT-associated parameters, transcription factors, and stem cell indices were assessed using immunoblot. The migration and self-renewal capabilities of cells were evaluated by wound healing and tumor sphere assays. The findings indicated that chrysin was effective in HeLa cell by inhibiting EMT and CSLC properties, NF-κBp65, and Twist1 expression [164].

#### Choriocarcinoma

Choriocarcinomas commonly progress by hydatidiform mole identified as overgrowth of tissue from the placenta or an abnormally fertilized egg, which is often observed in pregnant women. Choriocarcinomas possess the capability to metastasize quickly via a hematogenous route. Choriocarcinomas are commonly resistant to treatment and delayed diagnosis will make the curing process harder [165]. Inhibitory effects of chrysin on human choriocarcinoma cells (JAR and JEG3) was investigated by using proliferation assay (using the ELISA, BrdU kit), immunofluorescence microscopy (the expression of PCNA). FITC Annexin V apoptosis detection kit I, lipid peroxidation assay, mitochondrial staining kit (changes in the JC-1 mitochondrial membrane potential), Cytosolic calcium ion concentration assay and Western blot analysis (protein expression). Chrysin disrupted intracellular homeostasis by altering MMP, cytosolic Ca (2+) levels, ROS generation, and lipid peroxidation, which plays a role in the death of choriocarcinoma cells. As well as chrysin mediated the regulation of the AKT, ERK1/2, and JNK signaling pathways [166, 167]. Chrysin decreased 3*H*-2-deoxy-d-glucose apical uptake in human choriocarcinoma (BeWo) cells [168].

#### Lung cancer

Lung cancer is one of the causing reasons of carcinoma-connected fates [169]. The irregular expression of claudins (CLDNs), is observed in several solid tumors. CLDN1 and CLDN11 are significantly expressed in human lung squamous cell carcinoma (SCC). Chrysin decreased CLDN1 and CLDN11 expression in human lung SCC (RERF-LC-AI) cells. Chrysin alleviated p-Akt and inhibited PDK1 and Akt [170]. NiCl2 (Ni) induced migration and invasion in A549 and H1975 human lung cancer cells. Chrysin inhibited cytokines release, TNF-α, IL-1β, IL-10, and IL-6 induced by Ni in A549 cells. Chrysin suppressed TLR4 and Myd88 mRNA and protein expression. Furthermore, chrysin also decreased the nuclear level of p65 (NF-κB), the phosphorylation of IκB, and IKKβ, besides the MMP-9 expression in A549 cells exposed to Ni [171]. In a study of A549 cells conducted by Samarghandian and coworkers, chrysin increased Bax protein expression vice versa decreased Bcl-2 protein expression. Moreover, chrysin elevated caspase-3 and -9 activation confirmed the apoptotic role of chrysin on A549 cells [172]. Benzo (a) pyrene [B(a)P] induced lung carcinogenesis in mice was studied. Chrysin treatment mitigated lipid peroxidation and carcinoembryonic antigen and augmented CAT, SOD, GPx, GSH, GST, vitamin E, and vitamin C. Chrysin downregulated PCNA, COX-2, and NF-κB proteins expression [173]. In another study, chrysin activated AMPK in A549 cells [174]. Coadministration of chrysin and doxorubicin-induced apoptosis in A549 cells, H157, H1975, and H460 cells via modulation of MRP1, MRP3, and MRP5 expression and total GSH efflux [175]. Wang et al. demonstrated that chrysin could be used as a potential adjuvant therapy for drug-resistant NSCLC, especially for those with AKR1C1/1C2 overexpression [176]. 7-piperazine ethyl chrysin inhibited the viability of A-427 and A-549 lung cancer cells via suppression of ERK1/2 expression [169].

#### Pulmonary mucoepidermoid carcinoma

Mucoepidermoid carcinoma is the most frequent form of minor salivary gland malignancy in adults. Mucus secretion in the airway is a very main defense against microbial and chemical pollutants. Any abnormality in the production and secretion of mucins causes a pathological condition in the airway such as mucoepidermoid carcinoma. The effect of chrysin on NCI-H292 cells induced by PMA and EGF were evaluated by measuring MUC5AC mucin gene expression and mucin protein generation using rtPCR and ELISA assay. It was found that chrysin inhibited MUC5AC mucin generation and gene expression in a human pulmonary mucoepidermoid carcinoma cell line (NCI-H292) which is exposed to phorbol 12-myristate 13-acetate (PMA) or epidermal growth factor (EGF). The study found that chrysin inhibited the expression of mucin gene and mucin protein generation by direct effect on airway epithelial cells [177].

#### Anaplastic thyroid cancer

Anaplastic thyroid cancer (ATC) is a very aggressive thyroid gland malignancy with a very underprivileged prognosis [178, 179]. The effect of chrysin as a Notch activator was evaluated on ATC both in vitro and in vivo. Chrysin treatment upregulated mRNA levels of Notch1 and Hes1 (hairy/enhancer of split 1), a downstream Notch1 effector. Activation of Notch1 in vivo was related to the induction of cleaved PARP protein, representing that the growth inhibition was attributable to apoptotic cell death. Chrysin inhibited tumor growth in ATC both in vitro and in vivo through inducing Notch1 [180]. Chrysin inhibited growth and induced programmed cell death of ATC cells. Indeed, chrysin pretreatment led to Notch-1 activation and SLUG inactivation [181]. Phan and coworkers investigated the effects of chrysin on ATC (HTH7 and KAT18) cells. Cell proliferation was assessed each 48 h using MTT assay and western blot analysis was used for molecular indices of apoptosis. Pretreating cells with chrysin increased cleaved PARP, cleaved caspase-3, and declined cyclin D1, Mcl-1, and XIAP. Also, expression of the Bax/Bcl-2 ratio in ATC cells was augmented after chrysin exposure. The results suggested that chrysin was suitable for clinical for treating patients with ATC [182]. Wei et al. found that iodo-chrysin derivatives had higher anti-tumor effects on SW-579 and TT cell lines [183].

#### Nasopharyngeal carcinoma

Nasopharyngeal carcinoma (NPC) is the most frequent tumor in the nasopharynx in children and adults. Tumor necrosis factor (TNF)-related apoptosis-inducing ligand (TRAIL) belongs to the TNF family that can stimulate apoptosis in various cancer cells including NPC cells without affecting the human healthy cells. Pre-treatment of human cancer cell lines with chrysin induced cell death through stimulating TRAIL, as evidenced by the morphological alterations and present of sub-G1 peak. In HCT-116 cells, flow cytometry indicated that the percentage of sub-G1 increased with chrysin. It was observed that pretreatment with chrysin increased TRAIL-degraded caspase 3, caspase 8, and PARP proteins in human nasopharyngeal (CNE1 and -2) cells. Collectively, chrysin could stimulate apoptosis induced by TRAIL, and apoptosis is associated with caspase 8 activation [28, 184].

#### Melanoma

Melanoma is the most fatal skin cancer with poor prognosis and the global incidence of melanoma has increased in the recent 20 years [185, 186]. Human melanoma cancer A375.S2 cells were assessed regarding chrysin antimetastasis effects. Chrysin inhibited migration and invasion of A375.S2 cells that were examined by wound healing and the Transwell filter. Chrysin inhibited p-AKT (Thr308), PKC, GRB2, SOS-1, NF-κBp50, and NF-κBp65 expression, decreased Ras, PI3K, Snail, and p-c-Jun, p-AKT(Ser473), uPA, N-cadherin and VEGF, MMP-1, and MMP-2 [187]. In vitro and in vivo analysis were conducted by B16F10 cells and mice received B16F10. Chrysin administration inhibited cancer cell growth by inducing apoptosis and cell cycle arrest at the G2/M phase. Besides, chrysin treatment augmented the cytotoxic doings of CTL, NK, and macrophages [188]. A375 cells were used and chrysin showed in vitro anti-cancer activity that is allied with possible conscription of STAT-1, 3, 5 proteins at STAT (− 692 to − 684) region of p21 promoter and the induction of histone hyperacetylation. These findings also supported a surprising impact of chrysin on the chromatin organization of p21WAF1 promoter via hyper-acetylation and histone methylation [189]. Chrysin ameliorated TRAIL-mediated apoptosis in the SK-MEL-37 cell line [81]. Mouse melanoma cell line (B16-F1) and A375 cell lines were studied by Pichichero and coworkers. Pretreating cell lines with chrysin increased PBG-D expression. Besides, it increased caspase-3 and Bax and downregulated ERK 1/2, and activated of p38 MAPKs. Arrest in the G0/G1 phase of the cell cycle was also observed afterward chrysin treatment [190, 191]. Nano-encapsulated curcumin-chrysin, decreased expression of MMP-2, MMP-9, and TERT genes and increased expression of TIMP-1 and TIMP-2 genes in mouse B16F10 melanoma tumour model [192].

#### Uveal melanoma

Uveal melanoma is the most prevalent intraocular malignancy in adults. Human uveal melanoma cell lines (M17 and SP6.5) were used. Chrysin effect on cell viability and apoptosis, mitochondrial permeability were evaluated by using 3-(4,5-dimethylthiazol-2-yl)-2,5-diphenyltetrazolium bromide and terminal deoxynucleotidyl transferase mediated dUTP nick end-labeling tests and JC-1 fluorescein, respectively. Enzyme-linked immunosorbent assay was used for determine cytosol cytochrome c levels, and the activities of caspase-3, -8 and -9. Pretreatment with chrysin augmented mitochondrial permeability, cytosol cytochrome c levels, and caspases‑9 and ‑3 activities in M17 and SP6.5 cells. The findings of this study indicated that chrysin induced apoptosis of human uveal melanoma cells through the mitochondrial signaling pathway and recommended that chrysin might be a talented alternative neutraceutical in the management of uveal melanoma. It was found that chrysin caused apoptosis in human uveal melanoma cells through affecting the mitochondrial signaling [193].

#### Glioma

Glioma is the most corporate tumor of the CNS in adults [194, 195]. The effect of chrysin on glioblastoma cell lines and U87 xenografts in nude mice were studied. Cell proliferation was performed by using cell counting Kit-8 and a plate colony formation assay. Wound-healing test was used for measuring the ability of cell migration. The migration and invasion potential of cells were determined by Transwell migration and Matrigel invasion assay. Western blotting and immunofluorescence staining were used for assessing protein expression. Chrysin deactivated the Nrf2 signaling pathway by declining Nrf2 nuclear translocation and abrogating HO-1 and NQO-1 expression. Also, chrysin downregulated p- ERK1/2, protein expression however did not significantly alter p-P38 and p-JNK expression levels [196, 197]. It was suggested that chrysin induced anticancer activity in glioblastoma cells through the ERK/Nrf2 signaling.

Weng et al*.* evaluated the effectiveness of pretreatment with chrysin in rat C6 glioma cells by using cell viability, flow cytometric and western blotting analysis. Chrysin attenuated Rb phosphorylation and caused G1 phase cell cycle arrest. Besides, Chrysin mitigated CDK2 and 4 CDK4 activities and inhibited proteasome activity. Chrysin also induced p38-MAPK activation, resulting in the accumulation of p21 (Waf1/Cip1) protein or arbitrating the inhibition of proteasome activity [198].

#### Carcinoids

Carcinoids are neuroendocrine neoplasms characterized by significant rates of morbidity and mortality by the reason of a lack of effective therapeutic agents. Carcinoid cell lines (BON and H727) were utilized. The effect of chrysin on ASCL1 in carcinoid cell was evaluated by using western blotting. propidium iodide and phycoerythrin AnnexinV/7-aminoactinomycin D staining and sorting. Pretreatment with chrysin exposed S/G2 phase arrest and apoptosis. Interestingly, chrysin-induced cleavage of caspase-3 and PARP and activation of p21Waf1/Cip1. Direct ASCL1 knockdown with an ASCL1-specific, alleviating cyclin B1 and D1 and augmenting expression of p27Kip1 and p21Waf1/Cip1 was confirmable involved mechanisms to combat carcinoids. The findings suggested that inhibitory effect of chrysin on ASCL1 was effective for carcinoid management [199].

#### Leukemia

Chronic lymphocytic leukemia (CLL) progresses attributable to inequality among apoptosis and proliferation of B lymphocytes. B-CLL cells comprising JVM-13 and MOLT-4 cell lines were investigated. Exposure to chrysin induced activation of Bax and alleviated the expression of Bcl-2 protein, cytochrome c release from mitochondria into the cytosol, and cleaved/activated caspase-3, subsequently leading to the activation of apoptosis of B-CLL cells [200]. Chrysin treatment of CLL B-lymphocytes led to an increase in, ADP/ATP ratio, mitochondrial membrane potential collapse, the formation of ROS, activation of caspase 3, and apoptosis. Chrysin selectively inhibited ATPases and complex II in carcinomatous mitochondria as well [201]. Chrysin increased CD19 (B-cell marker), CD3 (T-cell marker), and Mac-3 (macrophages) cell surface markers and phagocytosis in treated WEHI-3 leukemic mice. Isolated splenocytes from chrysin-administered leukemic mice revealed an intensification of NK cell cytotoxicity [202]. Treatment of T-cell lymphocytes Jurkat cells with chrysin resulted in augmentation of cells proportion in the sub-G0/G1 phase of the cell cycle, which is reflected to be a marker of apoptotic cell death [203]. Lee and coworkers indicated the potent effects of chrysin in MO7e cell proliferation. Chrysin activated ERK5 and accelerated its translocation into the nucleus, and activated CREB and STAT3. Chrysin inhibited cell proliferation via inhibition of the PI3K pathway through SCF/c-Kit signaling and Shc/PDK1/PKC/Akt/c-raf signaling cascade [204]. Woo and coworkers utilized U937 cells, chrysin pretreatment activated Bcl2, and caspase-3 induced and PLC-gamma1 degradation. The stimulation of apoptotic cell death was also gone along with the down-regulation of X-linked inhibitor of apoptosis (XIAP) and the abrogation of Akt [205]. 8-bromo-7-methoxychrysin in combination with arsenic trioxide induced apoptosis in U937, HL-60, and Jurkat cells. This co-treatment led to cytochrome c release, down-regulation of XIAP and Bcl-XL, and up-regulation of Bax. Moreover due to this co-treatment declined Akt phosphorylation in addition to intracellular GSH content [206].

#### Osteoblast tumor

Osteoblasts are necessary for bone homeostasis via deposition of new bone osteoid into resorption pits. Osteoblasts have the main role in cancer cell propagation to bone and metastasis.

It was found that chrysin caused an increase in ROS production in osteoblasts UMR106 tumor cells which might lead to antitumor effects that detected by EPR spectroscopy [207].

#### Lymphangiogenesis

Lymphangiogenesis stimulation is the main process for inducing cancer growth and metastasis. Prevention of lymphangiogenesis is the main strategy for cancer therapy [208]. Proliferation assay, cord formation assay, adhesion assay and migration assay were used to determine the efficacy of chrysin in TR-LE cells. Inhibitory effect of chrysin on lymphangiogenesis was responsible for protective effect in TR-LE cells. Chrysin significantly inhibited cord formation, cell adhesion, and migration in rat lymphatic endothelial cells (TR-LE) through inducing VEGF-C mRNA expression and NO production [209].

#### Angiogenesis

Unregulated angiogenesis occurs in pathological states including cancer. STAT3 has a main role in the angiogenesis and inflammation processes in tumor metastasis. Chrysin suppressed IL-6-induced angiogenesis in human umbilical vein endothelial cells (HUVECs) and in ovo model of chicken chorioallantoic membrane assay through modulation of the soluble IL-6 receptor /gp130/JAK1/STAT3/VEGF signaling pathway [210].

Chrysin could inhibit angiogenesis through decrease in the expression of VEGF and IL-6 and also expression of their receptor in HUVECs exposed to lipopolysaccharide (LPS). Indeed, chrysin was able to suppress tumor progression through inhibition inflammatory molecules that involved in angiogenesis [211].

#### Ehrlich ascites carcinoma

Ehrlich ascites tumor (EAT) cells were utilized by Orsolić et al. Pretreating with chrysin reduced tumor size and total number of cells in peritoneal cavity of mice affected EAT. Besides, chrysin could increase the marine’s survival time and macrophage stimulation [212]. The findings indicated that the anti-tumor effect of chrysin in EAT cells was the results of related to increase in macrophages activity.

**Fig. 3:**
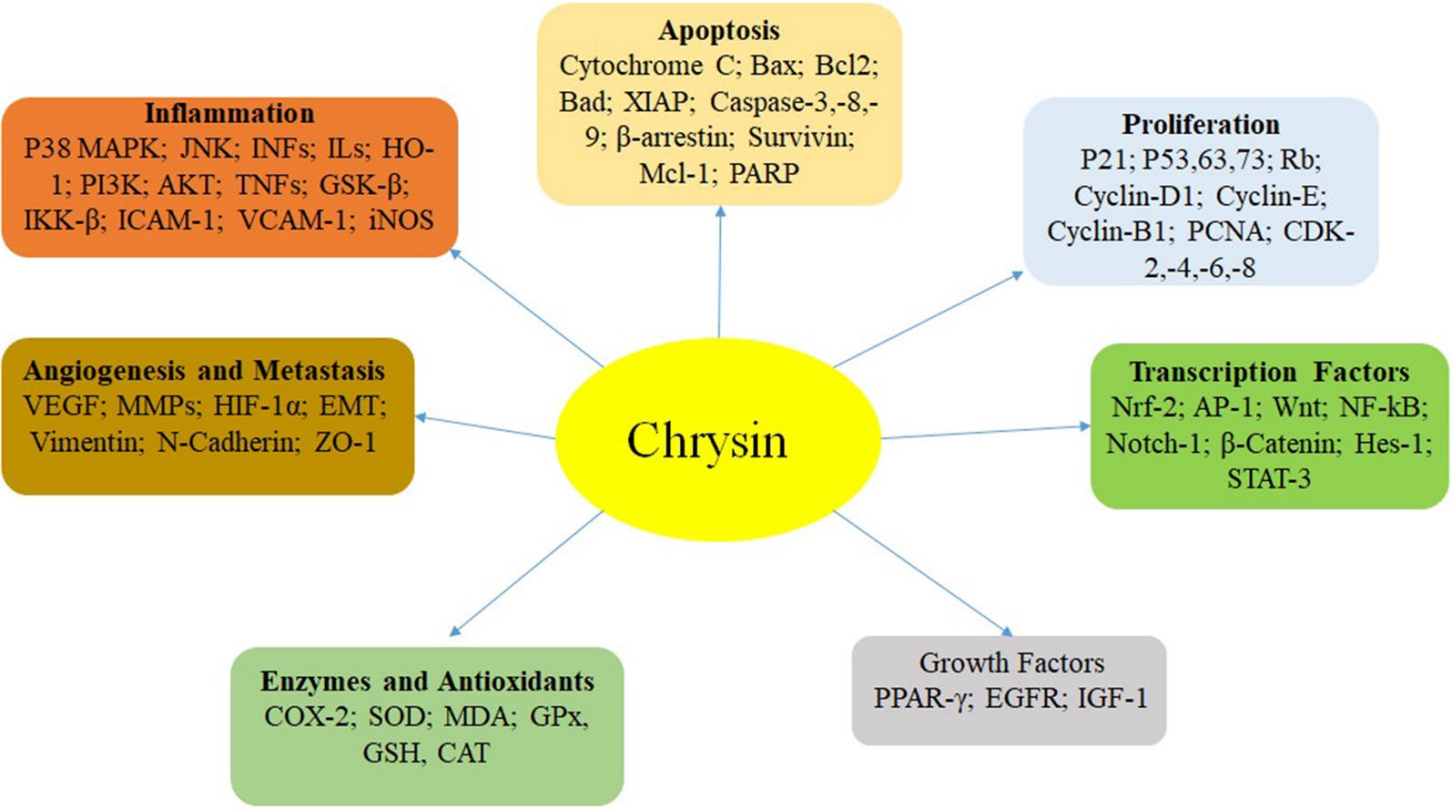
Important mechanisms involved in the anti-cancer activities of chrysin

Figure 3 indicates the important mechanism underlying the anti-tumor effects of chrysin.

## Conclusions and future challenges

Regarding the broad indications of chrysin in numerous clinical complications, we have defined the current study. Cellular and molecular mechanisms underlying therapeutic applications of chrysin in various cancers have been gathered and discussed. Oxidative stress, inflammatory responses, autophagy, and apoptosis were the most common mechanisms that were affected by chrysin. Chrysin could ameliorate cancers of the breast, gastrointestinal tract, liver and hepatocytes, bladder, male and female reproductive systems, choroid, respiratory tract, thyroid, skin, eye, brain, blood cells, leukemia, osteoblast, and lymph. Likewise, due to the low bioavailability of flavonoids such as chrysin, some modifications comprising a synthesis of analogs, design novel drug delivery systems, and using various carriers would be helpful.

## Data Availability

All data are available in the manuscript.
